# High prevalence of undiagnosed diabetes and abnormal glucose tolerance in the Iranian urban population: Tehran Lipid and Glucose Study

**DOI:** 10.1186/1471-2458-8-176

**Published:** 2008-05-24

**Authors:** Farzad Hadaegh, Mohammad Reza Bozorgmanesh, Asghar Ghasemi, Hadi Harati, Navid Saadat, Fereidoun Azizi

**Affiliations:** 1 Prevention of Metabolic Disorders Research Center, Research Institute for Endocrine Sciences, Shahid Beheshti University (M.C), Tehran, Iran

## Abstract

**Background:**

To estimate the prevalence of diagnosed and undiagnosed diabetes mellitus, impaired fasting glucose (IFG), impaired glucose tolerance (IGT), and combined IFG/IGT in a large urban Iranian population aged ≥ 20 years.

**Methods:**

The study population included 9,489 participants of the Tehran Lipid and Glucose Study with full relevant clinical data. Age-standardized prevalence of diabetes and glucose intolerance categories were reported according to the 2003 American Diabetes Association definitions. Age-adjusted logistic regression models were used to estimate the numbers needed to screen (NNTS) to find one person with undiagnosed diabetes.

**Results:**

The prevalence of diagnosed and undiagnosed diabetes, isolated IFG, isolated IGT, and combined IFG/IGT were 8.1%, 5.1%, 8.7%, 5.4% and 4.0% in men and 10%, 4.7%, 6.3%, 7.6%, and 4.5% in women respectively. Participants with undiagnosed diabetes had higher age, body mass index (BMI), waist circumference, systolic and diastolic blood pressures, triglycerides (all p values <0.001) and lower HDL-cholesterol (only in women, p < 0.01) compared to normoglycemic subjects. Undiagnosed diabetes was associated with family history of diabetes, increased BMI (≥ 25 kg/m^2^), abdominal obesity, hypertriglyceridemia, hypertension and low HDL-cholesterol levels. Among men, a combination of increased BMI, hypertension, and family history of diabetes led to a NNTS of 1.6 (95% CI: 1.57–1.71) and among women a combination of family history of diabetes and abdominal obesity, yielded a NNTS of 2.2 (95% CI: 2.1–2.4).

**Conclusion:**

In conclusion, about one third of Tehranian adults had disturbed glucose tolerance or diabetes. One- third of total cases with diabetes were undiagnosed. Screening individuals with BMI ≥ 25 kg/m^2 ^(men), hypertension (men), abdominal obesity (women) and family history of diabetes may be more efficient.

## Background

The number of people with type 2 diabetes mellitus is increasing worldwide [1] and many of these individuals remain unidentified [2]. Undiagnosed type 2 diabetes and impaired glucose regulation are reported to have substantial clinical importance [3,4] and increase the risk of cardiovascular morbidity and mortality [5,6]. Undiagnosed diabetes may also impose substantial public health implications because these subjects remain untreated and at risk for complications [2]. Although screening for undiagnosed diabetes within general practice by measuring fasting blood glucose is feasible but it would be best targeted at individuals with multiple risk factors for diabetes [7].

Since limited data are available on the Iranian population regarding prevalence of diabetes and other glucose tolerance abnormalities [8,9], this study aimed to determine the age and gender-specific prevalence of undiagnosed and diagnosed diabetes, impaired fasting glucose (IFG), impaired glucose tolerance (IGT), and combined IFG/IGT in a large urban population of Iranian adults in Tehran. We also assessed, the number needed to screen (NNTS), to identify one person with undiagnosed diabetes, and characteristics of individuals that might be most effectively targeted for screening programs.

## Methods

### Data collection

#### General information

The Tehran Lipid and Glucose Study (TLGS) is a longitudinal study, conducted to determine the risk factors for non-communicable diseases among the Tehranian population [10]. Briefly, 15,005 people aged 3 years and over, living in district 13 of Tehran, representative of the capital city, were selected by multistage cluster random sampling methods in the cross-sectional phase 1 of the TLGS from 1999 to 2001. The ethical committee of the research institute for endocrine sciences of Shahid Beheshti University (M.C) approved design of the study and informed written consent was obtained from participants older than 15 years, and from their parents if they were younger. In this study, we included participants aged ≥ 20 years (n = 10,368), if they did not report having diabetes according to a physician diagnosis and were not currently taking insulin or oral anti-diabetic agents (diagnosed diabetes); also individuals were excluded from the analysis if they had a missing fasting (*n *= 290) or 2-hours glucose value (*n *= 589) Finally, 9,489 individuals (men to women ratio 4,006:5,483), aged ≥ 20 years with valid data on glucose tolerance, were included in the current data analysis. The overall response rate was 91.5%. Demographic information was obtained by use of a standard and pretested questionnaire. Subjects were questioned about past and family (parents and siblings) history of diabetes mellitus and or taking of any anti-diabetic drugs.

#### Measurement of Clinical variables

A detailed description of the methods for measuring anthropometric variables including weight, height and waist circumference (WC) has been previously reported [10]. Body mass index (BMI) was calculated as weight in kilograms divided by the height in meters squared. Blood pressure was measured twice in a sitting position after 15 minutes rest and the mean of the two measurements was considered as the participant's blood pressure.

#### Laboratory measurements

After all participants had fasted for 12–14 hours overnight, blood samples were drawn between 7:00 to 9:00 and centrifuged within 30–45 min of collection. A Sellectra 2 auto-analyzer (Vital Scientific, Spankeren, Netherlands) was used in the TLGS research laboratory. Fasting plasma glucose (FPG) was measured by enzymatic colorimetric method with glucose oxidase technique. For 2h-OGTT, 75 g glucose was administrated orally and plasma glucose was measured 2 hours later (2h-PG). For serum lipid measurements, total cholesterol and triglycerides (TGs) kits (Pars Azmoon Inc., Iran) were used. Using enzymatic colorimetric tests, we assayed TGs with glycerol phosphate oxidase. High-density lipoprotein cholesterol (HDL-C) was measured after precipitation of the lipoprotein B containing lipoproteins with phosphotungstic acid. Inter- and intra- assay coefficients of variation (CV) for fasting and 2-hour glucose were 2.2%. Inter- and intra-assay CVs were 2% and 0.5% for HDL-C and 1.6% and 0.6% for TGs, respectively.

#### Definition of terms

Low leisure time physical activity was defined as exercising less than three times per week. Increased BMI was defined as ≥ 25 kg/m^2^, hypertension as systolic blood pressure ≥ 140 and/or diastolic blood pressure ≥ 90 mmHg, hypertriglyceridemia as TGs > 2.8 mmol/l and low HDL-C levels as HDL-C< 0.9 mmol/l [11]. Abdominal obesity was defined as waist circumference >102 (men) and > 88 cm (women) [12]. According to the 2003 ADA diagnostic criteria, people without previously diagnosed diabetes were categorized as follows: Normal glucose tolerance (NGT), FPG < 5.6 and 2h-PG <7.7 mmol/l; undiagnosed diabetes, FPG ≥ 7.0 or 2h-PG ≥ 11.1 mmol/l; isolated impaired fasting glucose (IFG), FPG 5.6 to 6.9 and 2h-PG <7.7 mmol/l; isolated impaired glucose tolerance (IGT), 2h-PG 7.7 to 11.0 and FPG <5.6 mmol/l; and combined IFG and IGT (IFG/IGT) as FPG 5.6 to 6.9 and 2h-PG 7.7 to 11.0 mmol/l [13].

#### Statistical analyses

Separate analyses were carried out for each gender. Taking into account the multistage stratified cluster random sampling procedure, total and gender-specific prevalence (95% confidence intervals) of diagnosed and undiagnosed diabetes at the time of the study as well as prevalence of IFG, IGT and IFG/IGT were calculated. Sampling weights, which accounted for the unequal probabilities of selection resulting from the complex design and non-response adjustment factors based on Iranian census bureau data (1996) on age and gender, were incorporated to the estimation process. Age-and gender-specific crude prevalence were also directly standardized to the overall age distribution in the world population [14]. Differences between age and gender groups were tested using the Chi-Square test. Means (standard errors of mean) are presented for HDL-C values, anthropometric parameters and systolic and diastolic blood pressure. Values for TGs were log-transformed because of skewed distribution. Analysis of Covariance (ANCOVA) was used to assess significance of difference between normal individual (NGT) and individuals of 5 different glucose tolerance categories considering age as a covariate. Adjustment for multiple comparisons was done by the Bonferroni test. Controlling for age, we used logistic regression to determine the impact of potential risk factors on undiagnosed diabetes. In the multivariate model, all of the associated risk factors from age-adjusted analysis were included. Predictive marginals computed by logistic regression were used to estimate prevalence of undiagnosed diabetes in each risk factor group. NNTS, to identify one person with undiagnosed diabetes, was obtained as the inverse of the estimated prevalence of undiagnosed diabetes in each (univariate) and a cluster (multivariate) of risk groups [15]. Statistical significance was set at p < 0.05 and all values were two-sided. Statistical analysis was performed by the Statistical Package for Social Sciences, version 15.0 for windows (SPSS Inc., Chicago, IL).

## Results

A total of 9,489 subjects (men: 4,006, women: 5,483), mean age of 43.5 ± 14.5 years, were included in the current data analysis. Diagnosed diabetes was detected in 9.1% of individuals (n = 877), half of which (n = 438) were on anti-diabetic drugs, while the other half (n = 439) were not. Undiagnosed diabetes, isolated IFG, isolated IGT, and combined IFG/IGT were identified in 4.9%, 7.3%, 6.7%, and 4.2% of individuals respectively. Age-standardized estimated prevalence of various glucose tolerance categories was comparable to the crude sample-based prevalence (Table 1).

**Table 1:** Age and gender wise prevalence of different glucose intolerance categories among Tehranian adults.

Age group (years)	Study Population (n)	Isolated IFG (%)	Isolated IGT (%)	IFG/IGT (%)	Undiagnosed Diabetes (%)	Known Diabetes (%)
**Men**						
20–29	694	5.1	1.0	0.7	0.8	1.1
30–39	1054	9.8	4.4	1.7	1.6	2.8
40–49	775	11.1	6.9	4.6	5.2	6.9
50–59	605	11.2	7.0	7.3	9.3	14.7
60–69	637	8.5	8.2	9.4	8.6	20.3
≥ 70	241	7.1	11.7	6.2	14.9	19.1
Un-standardized	4006	9.1(8.2–10.0)	5.8 (5.1–6.5)	4.6 (4.0–5.2)	5.1 (4.4–5.8)	9.0 (8.1–9.9)
Age-standardized^a^	4006	8.7 (7.8–9.6)	5.4 (4.7–6.1)	4.0 (3.4–4.6)	5.1 (4.4–5.8)	8.1 (7.3–8.9)
						
**Women**						
20–29	1171	3.4	2.7	0.9	0.4	0.7
30–39	1464	5.4	6.9	2.6	2.1	3.0
40–49	1131	7.8	9.7	6.0	6.9	8.7
50–59	926	9.4	9.9	7.3	8.4	17.5
60–69	664	9.1	9.6	7.6	9.8	25.4
≥ 70	127	4.9	12.5	7.4	6.1	27.2
Un-standardized	5483	6.6 (5.9–7.3)	7.6 (6.9–8.3)	4.6 (4.0–5.2)	4.9 (4.3–5.5)	9.4 (8.6–10.2)
Age-standardized^a^	5483	6.3 (5.7–6.9)	7.6 (6.9–8.3)	4.5 (4.0–5.0)	4.7 (4.1–5.3)	10 (9.2–10.8)
						
**Total (95%CI)**						
Un-standardized	9489	7.7 (7.2–8.2)	6.8 (6.3–7.3)	4.6 (4.2–5.0)	5.0 (4.6–5.4)	9.2 (8.6–9.8)
Age-standardized^a^	9489	7.3 (6.8–7.8)	6.7 (6.2–7.2)	4.2 (3.8–4.6)	4.9 (4.5–5.3)	9.1 (8.5–9.7)

IFG: impaired fasting glucose, IFG/IGT: combined impaired fasting glucose and impaired glucose tolerance, IGT: impaired glucose tolerance, NGT: normal glucose tolerance.

a: age-standardization is based on world population 2000.

### Gender difference in prevalence of glucose abnormalities

The prevalence of undiagnosed diabetes as well as combined IFG/IGT did not differ by gender; IFG was more prevalent among men than women, (8.7% vs. 6.3% respectively, p < 0.001), whereas IGT was more prevalent among women than men (7.6% vs. 5.4% respectively, p < 0.001), (Table 1). The prevalence of diagnosed diabetes was higher in women (10.0%) than men (8.1%) (p = 0.0015). No significant difference was observed in the total prevalence of diabetes (diagnosed and undiagnosed) between men and women (13.2 vs. 14.7%, P = 0.3).

#### Age- and gender- specific prevalence of glucose abnormalities (standardized to the world population 2000)

The age-specific prevalence of diagnosed diabetes rose with age, up to the 7^th ^and 8^th ^decade of life in men and women respectively. The age-specific prevalence of undiagnosed diabetes rose with age in both genders (p for trends < 0.001); but there was some decrease in men aged 60 to 69 years and women aged ≥ 70 years. The prevalence of combined IFG/IGT increased with age up to 7th decade in both genders (p for trends < 0.001). Consistent increases in prevalence of IGT with age were also observed in both genders (p for trends < 0.001). Prevalence of IFG increased with age among women (p for trends <0.001) but not men (p for trends = 0.15) (Table 1). Among men, the proportion of undiagnosed diabetes relative to total diabetes remained the same (40%) up to the 8^th ^decade of life, when it increased to 70%. Among women, however, this proportion was the same as those observed among men up to the 6^th ^decade of life, when it began to decrease to 30% and 20% in the 7th and 8th decades of life respectively.

#### Cardiovascular risk factors and glucose tolerance

In both genders all cardiovascular risk factors (except for HDL-cholesterol) were higher among subjects with any glucose abnormality as compared to NGT (Table 2). Among men, age-adjusted mean HDL-C level did not change by glucose tolerance groups, while women subjects with diabetes (diagnosed and undiagnosed) and IFG/IGT had lower HDL-C levels than normoglycaemic subjects. Overall, undiagnosed diabetes cases had higher cardiovascular disease risk profiles than diagnosed diabetic subjects, with subjects with IFG, IGT and IFG/IGT falling somewhere between undiagnosed diabetic and normoglycaemic participants.

**Table 2:** Anthropometric and metabolic characteristics of participants by glucose tolerance categories.^a^

Variables	NGT/NFG	Isolated IFG	Isolated IGT	Combined IFG/IGT	Undiagnosed diabetes	Known diabetes
**Men**						
Age (years)	38.4 ± 1.01	43.7 ± 1.02†	50.4 ± 1.02†	53.7 ± 1.02†	54.2 ± 1.02†	56.1 ± 1.02†
BMI (kg/m^2^)	25.3 ± 0.1	26.3 ± 0.2†	27.0 ± 0.3†	27.4 ± 0.3†	28.0 ± 0.3†	26.6 ± 0.2†
WC (cm)	87.1 ± 0.2	90.0 ± 0.6†	91.7 ± 0.7†	93.1 ± 0.8†	94.4 ± 0.8†	91.0 ± 0.6†
SBP (mmHg)	119 ± 0.3	121 ± 0.9	125 ± 1.1†	128 ± 1.2†	131 ± 1.2†	126 ± 0.9†
DBP (mmHg)	76.9 ± 0.2	79.1 ± 0.6*	79.8 ± 0.7†	81.0 ± 0.8†	81.3 ± 0.8†	79.3 ± 0.6*
HDL-C (mmol/l)	1.0 ± 0.01	0.97 ± 0.01	0.99 ± 0.01	1.01 ± 0.01	0.98 ± 0.01	0.97 ± 0.01
TGs (mmmol/l)	1.61 ± 0.01	1.86 ± 0.01†	2.0 ± 0.01†	2.02 ± 0.01†	2.38 ± 0.01†	2.08 ± 0.01†
						
**Women**						
Age (year)	36.4 ± 1.01	44.7 ± 1.01†	45.4 ± 1.02†	48.8 ± 1.02†	51.1 ± 1.02†	54.0 ± 1.01†
BMI (kg/m^2^)	27.1 ± 0.1	28.8 ± 0.2†	28.3 ± 0.2†	29.4 ± 0.3†	29.4 ± 0.3†	27.7 ± 0.2
WC (cm)	86.2 ± 0.2	91.1 ± 0.6†	89.5 ± 0.5†	92 ± 0.7†	94.1 ± 0.7†	90.4 ± 0.5†
SBP (mmHg)	117 ± 0.3	121 ± 0.9*	122 ± 0.8†	126 ± 1.0†	128 ± 1.0 †	127 ± 0.8†
DBP (mmHg)	76.9 ± 0.2	79.3 ± 0.5†	80.4 ± 0.5†	80.5 ± 0.6†	82.1 ± 0.6†	79.7 ± 0.5†
HDL-C (mmHg)	1.18 ± 0.01	1.14 ± 0.01	1.14 ± 0.01	1.10 ± 0.01*	1.11 ± 0.01*	1.12 ± 0.01*
TGs (mmol/l)	1.43 ± 0.01	1.60 ± 0.01†	1.77 ± 0.01†	1.84 ± 0.01†	2.16 ± 0.01†	1.90 ± 0.01†

a: According to the 2003 ADA classification.

Data are means ± SEM (geometric mean ± SE for triglycerides).* p < 0.01, † p < 0.001: age-adjusted (except for age) comparison with combined normal glucose tolerance and normal fasting glucose (NGT/NFG). BMI: body mass index, DBP: diastolic blood pressure, HDL-C: high-density lipoprotein cholesterol, IFG: impaired fasting glucose, IGT: impaired glucose tolerance and IFG/IGT: combined impaired fasting glucose and impaired glucose tolerance, SBP: systolic blood pressure, TGs: triglycerides, WC: waist circumference.

#### Number needed to screen (NNTS) for undiagnosed diabetes

In the age-adjusted analysis, undiagnosed diabetes was associated with family history of diabetes, increased BMI, abdominal obesity, hypertriglyceridemia and hypertension in both genders and with low HDL-C levels only in women (Table 3). Increased BMI (OR; 95%CI: 4.1; 2.7–6.1) and abdominal obesity (2.9; 2.1–4.1) had the highest association with undiagnosed diabetes among men and women respectively. In multivariate analysis, increased BMI (3.3; 2.2–5.0), hypertension (2.2; 1.6–3.0), hypertriglyceridemia (2.3; 1.6–3.1) and family history of diabetes (2.1; 1.5–2.9) were associated with undiagnosed diabetes in men. The corresponding variables in women were abdominal obesity (2.5; 1.8–3.6), hypertriglyceridemia (2.6; 1.9–3.6) and family history of diabetes (2.0; 1.4–2.7).

**Table 3:** Risk factors associated with undiagnosed diabetes in the Tehranian adults.

Variables	Men		Women	
		
	Odds ratio^a^	95%CI	Odds ratio^a^	95%CI
Family history of diabetes (yes)	1.7^b^	1.2–2.3	1.8	1.4–2.3
Blood pressure > 140/90 mmHg	2.5	1.8–3.4	1.9	1.5–2.6
BMI ≥ 25 kg/m^2^	4.1	2.7–6.1	2.7	1.8–4.0
Waist circumference (cm) men: >102, women: >88	2.7	1.9–3.8	2.9	2.1–4.1
Low leisure time physical activity	0.82	0.5–1.5	1.0	0.7–1.6
Triglycerides > 2.8 mmol/l	2.4	1.8–3.3	2.8	2.2–3.7
HDL-C< 0.9 mmol/l	1.1	0.8–1.4	1.6	1.3–2.1

a: Odds ratio (95% confidence interval): age-adjusted univariate logistic regression analysis.

b: all p values <0.05 except for low leisure time physical activity in both gender and HDL-C in men. BMI: body mass index, CI: confidence interval, HDL-C: high-density lipoprotein cholesterol.

NNTS (95%CI) to identify one person with un-diagnosed diabetes in each risk factor groups is presented in Table 4. The lowest NNTS in men and women was observed for subjects with abdominal obesity and hypertriglyceridemia respectively. Based on the results obtained from multivariate regression model, a combination of family history of diabetes, increased BMI, and hypertension in men and family history of diabetes and increased WC in women led to the lowest calculated NNTS of 1.6 (95%CI: 1.57–1.71) and 2.2 (95%CI: 2.1–2.4) respectively.

**Table 4:** Number needed to screen for undiagnosed diabetes in various risk factor categories among the Tehranian adults.

Variables	Men		Women	
		
	NNTS^a^	95% CI	NNTS^a^	95% CI
Family history of diabetes (yes)	15.6	14.0–17.7	13.7	12.5–15.1
Blood pressure ≥ 140/90 mmHg	8.3	7.7–9.1	9.3	8.7–10.1
BMI ≥ 25 kg/m^2^	13.5	12.1–15.1	15.5	14.1–17.3
Abdominal obesity: waist circumference (cm)>102 in men and > 88 in women	8.0	8.4–8.7	12.5	11.5–13.8
Triglycerides > 2.8 mmol/l	10.8	9.8–11.9	7.8	7.3–8.4
HDL-C< 0.9 mmol/l	19.2	17.0–22.1	17.0	15.4–19.0

a: Number needed to screen (95% confidence interval) was obtained by univariate logistic regression analysis.

BMI: body mass index, CI: confidence interval, HDL-C: high-density lipoprotein cholesterol,

## Discussion

In a population-based study of Iranian urban residents, we reported prevalence of previously diagnosed and undiagnosed diabetes, isolated IFG, isolated IGT, and combined IFG/IGT, using OGTT. We found that about one-thirds of Tehranian adults aged ≥ 20 years were affected by some degrees of hyperglycemia. Approximately 14% of all participants were known to be involved with diabetes, of whom about one-third were undiagnosed.

The reported prevalence rate of diabetes in the current study was more than twice the rate predicted by King et al. for the Iranians in 2000. They estimated the prevalence of diabetes to be 5.5%, 5.7%, and 6.8% in 1995, 2000, and 2025 respectively [16]. As acknowledged, such studies are flawed with old data and are limited by paucity of data and assumptions required for generating the estimates [16]. Recently the national prevalence of type 2 diabetes among Iranian citizens, aged 25 to 64 years, has been reported to be 7.7%; of these half had undiagnosed diabetes [9]. It seems that the prevalence of diabetes and pre-diabetes states among Iranian adults are the same as those reported from neighbouring countries [17,18]. Similar to our results, the prevalence of diabetes using WHO criteria in an urban population in south India was 14.3% [19]. High prevalence of obesity and metabolic syndrome might contribute to an increased prevalence of glucose tolerance abnormalities in Iran [20-23]. Undiagnosed diabetes is reported to be as prevalent as or even more prevalent than diagnosed diabetes [24,25]. The proportion of undiagnosed relative to total diabetes has been reported to be 70% among the Danish [26], 60.6% in Indians [19] and 47% among Australians [27]. The proportion that was reported in the current study is in concordance with the results from the US population [28]. Although there is increased awareness of diabetes in our population with improvement of education and access to medical care, but this was not sufficient to decrease the percent of undiagnosed cases.

The association between diabetes and gender has been the focus of several studies with inconsistent results [29-32]. We found that the total prevalence of type 2 diabetes was higher in women than men (10 vs. 8.1), a finding that is confirmed by the national survey of diabetes in Iran (8.3% in women vs. 7.1% in men) [9] and differs from data of the U.S [33] and Australia [27]. The higher prevalence of metabolic syndrome in our women population may be the underlying cause for this sex difference [20,21]. In the current study, IGT was more prevalent among women, whereas IFG was observed more among men. Data from national survey of diabetes in Iran [9] and the U.S. [33] also showed the higher prevalence of IFG in the male population. It was previously shown that in populations where IFG was more prevalent in men, IGT was identified more among women [29]. In many studies, it was reported that the prevalence of diabetes increased with age [29,31]. In the current study, in both genders, prevalence of diagnosed and undiagnosed diabetes, IGT, and IFG/IGT increased with age, whereas IFG prevalence increased with age only in women

Undiagnosed type 2 diabetes is not milder than clinically detected diabetes [15]. We found that cardiovascular risk profile of subjects with undiagnosed diabetes either equalled or was higher than that of previously diagnosed patients. Hariss et al. in a review study reported that people with undiagnosed diabetes have substantial rates of risk factors for diabetes complications although they are not as hyperglycemic as are patients with diagnosed diabetes [34]. Similar to other reports [35,36], most risk factors independently associated with undiagnosed diabetes in the current study were components of the metabolic syndrome [12], high prevalence of which were reported in our population [20,21].

In the current study, we identified a combination of risk factors that had the lowest NNTS in our population. The target group for diabetes screening among men was distinct from women. Screening among men with family history of diabetes, increased BMI, and hypertension, and among women with family history of diabetes and abdominal obesity, is more efficient. In our multivariate analysis, we showed that high BMI in men and high WC in women were independent risk factors for type 2 diabetes, findings that were supported by the OBESITY in ASIA Collaboration study [37]. Like other reports [15], the current study showed that screening for type 2 diabetes might be more efficient among men due to lower NNTS. Our data as that of the Rathmann et al. [15] study, showed that hypertriglyceridemia, as a marker of insulin resistance, had the least NNTS in women compared to men (7.8 vs. 10.8 respectively); in multivariate regression analysis however it was excluded from our model; thus we presented a more practical model for identifying undiagnosed diabetes in both genders.

There are several points that should be considered when examining the results of this study. First, given the rising trend in the prevalence of obesity [23] and diabetes [38] in our population, the reported figures in this study might be an underestimation. Second, the cross-sectional study design prevents causal inference to be made about the relationship between risk factors and undiagnosed type 2 diabetes. The strengths of our study include using both FPG and 2h-PG for determining of undiagnosed diabetes and having a large sample size, representative of Tehran.

## Conclusion

In conclusion, about one third of Tehranian adults had disturbed glucose tolerance or diabetes. One- third of total cases with diabetes were undiagnosed. Screening individuals with BMI ≥ 25 kg/m^2 ^(men), hypertension (men), abdominal obesity (women) and family history of diabetes may be more efficient; however, further evidence is required to identify how, who, and how often people should be screened.

## Competing interests

The authors declare that they have no competing interests.

## Authors' contributions

FH participated in the conception and design of the study and its final approval. MRB drafted the manuscript and performed the statistical analysis. AG revised the manuscript for important intellectual content. HH drafted the manuscript and helped in statistical analyses. NS revised the manuscript for important intellectual content. FA participated in its design and coordination. All authors read and approved the final manuscript.

## Pre-publication history

The pre-publication history for this paper can be accessed here:


